# Ocular squamous cell carcinoma in Holstein cows from the South of Brazil

**DOI:** 10.14202/vetworld.2017.1413-1420

**Published:** 2017-12-01

**Authors:** Gabrielle A. Fornazari, Juliana Kravetz, Matti Kiupel, Dodd Sledge, Ivan Roque De Barros Filho, Fabiano Montiani-Ferreira

**Affiliations:** 1 Graduate School Program in Veterinary Sciences (PPGCV-UFPR), Federal University of Paraná, Rua dos Funcionários, 1540, 80035-050, Juvevê, Curitiba-PR, Brazil; 2 Veterinary Diagnostic Laboratory, 4125 Beaumont RD BLDG 0215, Room 152A, Lansing, MI 48910, USA

**Keywords:** bovine, histologic classification, ophthalmology, p16, p53, tumor behavior

## Abstract

**Aim:**

The aim of this study was to investigate 10 cases of bovine ocular squamous cell carcinoma (OSCC) diagnosed in Holstein or Holstein-crosses cows.

**Materials and Methods:**

The investigation was performed exclusively in OSCC cases diagnosed in the State of Paraná and Santa Catarina. A combination of two previously existing histopathological classifications systems was used. The tissue samples were tested for immunoexpression of p53 and p16 and polymerase chain reaction (PCR) for bovine herpesvirus and papillomavirus.

**Results:**

A positive correlation between number of mitotic figures and tissue invasion was found. Anaplasia parameters did not correlate well with tumor invasion of deeper tissues and mitotic counts. Six of 10 OSCC cases were in animals with heavily pigmented eyes. Immunoexpression of p53 and p16 was observed in 3 cases each. Bovine herpesvirus and papillomavirus were not detected by PCR.

**Conclusions:**

Our results indicate that OSCC occurrence is most likely multifactorial with genetic, phenotypic, and environmental influences contributing to the pathogenesis of the disease.

## Introduction

Ocular squamous cell carcinoma (OSCC or “cancer eye”) has been recognized in the literature since the latter part of the 19^th^ century [1]. It is a primary neoplasm of epithelial origin that may occur in different ocular and periocular tissues including the palpebral skin, epithelial surfaces of the cornea and conjunctiva, third eyelid, and limbus. OSCC occurs with high frequency in cattle all over the world [2], and it is the leading cause of enucleation among all other ocular diseases [3]. The reported incidence of OSCC in European countries is lower than in Africa and the Americas [4].

European breeds of cattle, mainly “taurine” (*Bos taurus*), cattle and their crosses – particularly those with unpigmented skin on the face – commonly develop OSCC [3]. OSCC occurs in other farm animal species including sheep, swine, goats, and horses but with a lower incidence [5].

In many countries, OSCC is considered the most common form of neoplasia or among the top three most frequently reported neoplastic diseases affecting cattle and has significant economic importance [1-8]. In the United States, the prevalence of OSCC varies with geographic distribution and is higher in the southwestern region and lower latitudes with higher levels of sunlight [9,10]. To date, there are no general epidemiologic studies or retrospective investigations of OSCC in cattle throughout Brazil. There are, however, some surveys from few Brazilian states including Pará [11] and Paraíba [12]. Other Brazilian studies reviewed the prevalence of bovine neoplasms that had been submitted to veterinary pathology laboratories [13] or the prevalence of bovine OSCCs among ocular neoplasms [14].

Squamous cell carcinomas (SCC) commonly have mutations in p53, and positive immunolabeling for p53 has been reported in humans and animals especially in SCCs of non-pigmented skin secondary to exposure to UV radiation [9,10]. SCCs have been shown to express p16 through immunolabeling. Antibodies targeting p53 and p16 have been used as prognostic factors in human cutaneous SCCs [15] although no relationship has been found in cattle with OSCC [16]. Viruses including bovine papillomavirus and herpesvirus have been shown to induce preneoplastic lesions as well as neoplastic transformation [17,18]. Papillomaviruses are well known to cause proliferative lesions of the epidermis in the skin and mucosal membranes that may undergo malignant transformation [19-22]. Nevertheless, Anson *et al*. [23] were not able to isolate bovine herpesvirus from 31 tissue homogenates of OSCC. They hypothesized that isolations of this virus previously published by other groups were actually from passenger viruses since the majority of reported herpesvirus tumors yield infectious virus only after induction by UV light, steroids, or pH changes [23].

The objective of this investigation was to study OSCC cases referred to the Laboratory of Comparative Ophthalmology (LABOCO), Federal University of Paraná (UFPR) exclusively from samples of affected Holstein and Holstein-crosses cows from the State of Paraná and Santa Catarina, in the south of Brazil and to characterize these case by a combination of histologic examination, immunohistochemical evaluation, and polymerase chain reaction (PCR) for viral detection.

## Materials and Methods

### Ethical approval

The Institutional Animal Care and Use Committee approved the research and, when necessary, a consent form was signed by the owners.

### Samples

Ten OSCCs from milk-producing cows were submitted to the LABOCO-UFPR between March 2013 and December 2014. All animals were Holstein and Holstein-cross breeds. Six samples (Bov12, Bov16, Bov21, Bov26, Bov27, and Bov30) were collected by a large animal veterinary practitioner working in the city of Guarujá do Sul, State of Santa Catarina, Brazil (26°23’07”S 53°31’40”W). All these cases from Guarujá do Sul were surgically managed by excisional biopsies. One sample (Bov09) also was surgically managed by excisional biopsy by one of the authors (FMF) at the Canguiri Experimental Farm owned by UFPR and located at the city of Quatro Barras in the State of Paraná, Brazil (25º21’56”S 49º04’37”W). Three samples (Bov4, Bov15, and Bov17) were donated by an abattoir (Argus Ltd., SIF 1710) located at the city of São José dos Pinhais in the State of Paraná, Brazil (25°32’06”S 49°12’21”W) (Table-1).

**Table-1:** Signalment and clinical signs of cows with ocular squamous cell carcinoma.

Case ID	Age (years)	Lesion localization	Affected eye	Tumor surface appearance	Eyelid color	Breed	Origin
Bov04	3	Corneo conjunctival	OD	Congested round plaque	Unpigmented	Holstein cross	PR
Bov09	7	Third eyelid	OD	Verrucous, hyperemic (with purulent discharge)	Pigmented	Holstein	PR
Bov12	4	Third eyelid	OS	Small nodules, hyperemic	Pigmented	Holstein	SC
Bov15	3	Corneo conjunctival	OD	Congested plaque	Unpigmented	Holstein cross	PR
Bov16	6	Inferior eyelid	OS	Hyperemic plaques, multiple crusty lesions	Pigmented	Holstein	SC
Bov17	3	Corneal	OS	Verrucous mass	Unpigmented	Holstein cross	PR
Bov21	8	Third eyelid	OD	Small nodules, congested, purulent discharge	Unpigmented	Holstein cross	SC
Bov26	6, 7	Third eyelid	OS	Nodular, congested	Pigmented	Holstein	SC
Bov27	3	Third eyelid	OD	Nodular, congested	Pigmented	Holstein	SC
Bov30	5	Third eyelid	OD	Nodular with verrucous surface, congested	Pigmented	Holstein	SC

OD=Right eye, OS=Left eye, SC=Santa Catarina state, PR=Paraná state

### Histopathological and immunohistochemical processing and grading

Samples were fixed in 10% formalin, embedded in paraffin, and 5 µm serial sections were cut. One section of each case was stained with hematoxylin and eosin for routine histopathological examination. All OSCCs were classified according to clinical information (when available), macroscopic, and microscopic features (Tables-1 and 2). OSCCs were graded according to the Broder’s system based on the degree of keratinization and island formation of neoplastic cells [24,25] as well as according to the classification proposed by Carvalho *et al*. [16] centered on cellular differentiation, mitotic count, and degree of invasion. Based on these criteria, the following scores would be assigned to each OSCC: A minimal anaplasia score (1) would be assigned to well-differentiated OSCCs characterized by neoplastic cells with abundant cytoplasm that commonly formed concentric laminated masses of keratin (keratin pearls); a moderate anaplasia score (2) would be assigned to OSCCs that had a moderate degree of keratinization, small- to medium-sized keratin pearls, smaller islands of neoplastic cells, and an increased number of poorly differentiated cells; and a maximal anaplasia score (3) would be attributed to neoplasms consisting of poorly differentiated cells that did not form keratin pearls and only had individual cells undergoing keratinization. Tissue invasion was classified based on the extension into adjacent tissues: (1) None to minimum invasion; (2) neoplastic cells extending into immediately adjacent tissues (i.e., eyelid dermis, corneal stroma, and conjunctival substantia propria); and (3) neoplastic cells extending into deeper tissues (i.e., muscle, uvea). The mitotic cell figures (MCF) count were analyzed in 10 high-power fields (400×) (hpfs), averaged, and expressed as MCF/hpf. The OSCCs were classified calculating the sum of these three scores: Anaplasia, invasion and mitotic figures count, and creating an “AIM” score. In addition, the presence and degree of the inflammatory infiltrate were evaluated and scored as well.

**Table-2:** Results of the proposed tumor grading according to selected histopathological features and immunohistochemical staining.

Case ID	Degree of anaplasia	Tissue invasion	Mitotic figures (hpf)	Sum “AIM” score	Inflammation	p53 LI (%)	Nuclear p16	Cytoplasmic p16
Bov04	2	1	0.8	3.8	1	10	-	-
Bov09	1	2	0.9	3.9	2	15	-	+
Bov12	3	1	0.5	4.5	2	0	-	-
Bov15	1	3	2.6	6.6	1	0	-	-
Bov16	1	2	2.2	5.2	1	0	-	+
Bov17	2	2	3.8	7.8	1	29	-	-
Bov21	1	2	2.2	5.2	2	0	-	+
Bov26	2	1	0.7	3.7	2	NA	NA	NA
Bov27	2	2	0.9	4.9	3	NA	NA	NA
Bov30	1	2	2.4	5.4	1	0	-	-

hpf=High power field, LI=Labeling index, AIM=Anaplasia+invasion+mitosis

## Immunohistochemistry

Eight of ten paraffin-embedded samples were submitted to Michigan State University for immunohistochemical evaluation for p53 and p16. Deparaffinization, antigen retrieval, and immunolabeling were performed on automated immunostainers. Immunohistochemical labeling for p16 was performed on the Bond maX^™^ Automated Staining System (Vision BioSystems^™^) using the Bond^™^ Polymer Detection System (Vision BioSystems^™^) and a mouse monoclonal antibody against p16 (clone 6H12, NovoCastra^™^) at a dilution of 1:20. Antigen retrieval was achieved using the Bond Epitope Retrieval Solution 2 (Vision BioSystems^™^) for 20 min. The immunoreaction was visualized with 3,3-diaminobenzidine substrate (Vision BioSystems^™^), and sections were counterstained with hematoxylin. Immunohistochemical labeling for p53 was performed on the Bench Mark Automated Staining System (Ventana Medical Systems, Inc.) using the Enhanced V Red Detection (Alk. Phos. Red) Detection System (Ventana Medical Systems, Inc.) and a rabbit polyclonal antibody against p53 (Signet Laboratories) at a dilution of 1:100. Antigen retrieval was achieved using the Ventana Medical Systems Retrieval Solution CC1 (Ventana Medical Systems) for 60 min. Sections were counterstained with hematoxylin. Positive immunohistochemical controls included a canine soft tissue sarcoma with strong p53 expression and bovine esophagus to which the appropriate antibodies were added. For negative controls, the primary antibodies were replaced with buffer. Only nuclear labeling was evaluated as a positive signal for p53, while cytoplasmic and nuclear labeling was recorded for p16. OSCCs were divided into positive or negative labeled tissues based on the absence or expression of p16 and p53, respectively. Furthermore, using the labeling index (LI) proposed by Kato *et al*. [26] 500 cancer cell nuclei were observed in the areas of the sections with highest labeling frequencies and then considered as focal/weak if <25% of cells were positive or showed only blush positivity, and positive if strong positivity was seen in ≥25% of cells at 100× magnification. Staining of <5% was considered negative expression.

### In situ hybridization (ISH)

ISH for papillomavirus was performed using the Discovery automated slide-processing system (Ventana Medical Systems, Inc.) as previously described [27]. Briefly, slides received pre-treatment through mild cell conditioning with the citrate buffer based RiboCC reagent (Ventana Medical Systems, Inc.) and enzyme pre-treatment with Protease #3 for 12 min (Ventana Medical Systems, Inc.). Sections were hybridized with a generic antisense probe for papillomaviruses (5’-/5DigN/TGG TTY TKY SYT RWV MGD GAR CAR ATG TWT GYY MGD CAT WTG TGG-3’) at 200 ng/slide for 1 h at 37°C after a denaturing step for 4 min at 95°C. Three stringency wash steps with 0.5× Ribo-Wash (Ventana Medical Systems, Inc.; equivalent to 0.5× saline sodium citrate) for 4 min at 42C were followed by incubation with an anti-rabbit anti-digoxigenin antibody (Sigma) for 32 minutes at 42°C. After incubation with streptavidin-alkaline phosphatase conjugate UMap anti-Rb AP (Ventana Medical Systems, Inc.) for 16 min at 42C, labeled viral DNA was visualized with the BlueMap NBT/BCIP substrate kit (Ventana Medical Systems, Inc.) for 2 h at 42C. Finally, sections were counterstained with nuclear fast red equivalent reagent Red Stain II (Ventana Medical Systems, Inc.) for 4 min before coverslipping.

### PCR for generic herpesvirus and generic papillomavirus

Eight of ten samples were submitted for PCR for generic herpesvirus and PCR and ISH for generic papillomaviruses to Michigan State University. Shavings of paraffin-embedded formalin-fixed OSCCs were submitted for DNA extraction using the DNeasy Tissue Kit (QIAGEN, Valencia, CA), following the manufacturer’s protocols. A set of degenerate PCR primers that amplify 21 species of herpesviruses (8 human and 13 animal viruses) [28] were used to detect the presence of herpesvirus. 25 microliter of PCR mixture contained 5.0 µL (1-750 ng) of template DNA, 1 µM of each primer (5’-TGTAACTCGGTGTAYGG NTTYACNGGNGT-3’ and 5’-CACAGAGTCC GTRTCNCCRTADAT-3’), 80 µM (each) of deoxynucleoside triphosphate, 0.5U of Taq polymerase (Invitrogen, Carlsbad, CA), 2.5 µL of 10× PCR buffer (200 mM Tris HCl (pH 8.4) and 500 mM KCl, Invitrogen, Carlsbad, CA), and 2 mM MgCl_2_. The PCR reaction was performed under mineral oil and cycled 45 times with 30 s of denaturation at 94°C, 1 min of annealing at 46°C and 1 min of extension at 72°C. DNA was also tested by PCR using two previously reported assays for the detection of a broad range of human papillomaviruses. Both assays were performed using the QIAGEN Taq PCR Master Mix Kit. The first assay targets a 450 bp region of the papillomavirus L1 gene with primers MY11 (5’-GCMCAGGGWCATAAYAATGG-3’) and MY09 (5’-CGTCCMARRGGAWACTGATC-3’) [29], each at 0.5 µM final concentration in a 50 µl reaction volume. PCR was carried out in 40 cycles of 94°C for 30 s, 53°C for 30 s, and 72°C for 30 s. The second assay amplifies either a ~450 bp (primers CP4, 5’-ATGGTACARTGGGCATWTGA-3’ and CP5, 5’-GAGGYTGCAACCAAAAMTGRCT-3’) or a ~320 bp (primers PPF1,5’-AACAATGTGTA GACATTATAAACGAGC-3’ and CP5) region of the papillomavirus E1 gene [30]. Assay specifications were similar to a previous report [31] wherein the reaction was carried out in a multiplex format. The cycling conditions were adjusted to 40 cycles of 94°C for 30 s, 47°C for 30 s, and 72°C for 1 min followed by a final extension of 72°C for 7 min.

Viral isolates, as well as paraffin blocks containing tissues infected with the viral targets as previously demonstrated, were used as positive controls. PCR products were analyzed by agarose gel electrophoresis and visualized by UV transillumination. Detected amplicons were gel-purified using the QIAquick Gel Extraction Kit (QIAGEN) and were submitted for automated direct sequencing to the Research Technology Support Facility of Michigan State University. DNA sequence was analyzed by BLAST [32] to search for significant similarities to sequences in the GenBank database. Sequence assembly and editing were performed with the Lasergene biocomputing software (DNASTAR, Inc., Madison, WI).

### Statistical analysis

Pearson product-moment correlation coefficient was used to measure the linear correlation between the results of the analyzed samples. Descriptive and inferential statistics were performed using a computer program (StatView Software, Cary, NC). Pearson’s r was calculated and p<0.05 was considered significant.

## Results

**Figure-1:**
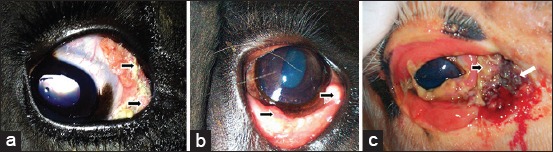
Representative examples of the clinical appearance of selected cases of ocular squamous cell carcinoma investigated. (a) Bov09 - Chronic third eyelid margin masses (arrows) in the right eye, which was discretely verrucous, hyperemic (with discrete purulent discharge). Note that the eyelid and uveal tissue were pigmented. (b) Bov12 - Small, hyperemic nodular lesions in the left third eyelid margin (arrows). Note that the eyelid and uveal tissue also are pigmented in this animal. (c) Bov21 - Small congested nodules in the right third eyelid (black arrow). Note the substantial purulent discharge due to local parasitic infestation by fly larvae (maggots) (white arrow). In this animal, the eyelid was not pigmented.

A summary of the clinical signalment and gross appearance of the 10 OSCCs is presented in Table-1. Six cows had pigmented (black) eyelids (Figure-1a and b), three were non-pigmented (white), and one was partially pigmented (white with dark patches) (Figure-1c). The mean age of the animals affected was 5 years; the minimum age was 3 years, and the maximum age was 8 years. Of the 10 OSCCs, six were present in the third eyelid, three in the corneo conjunctival junction (*limbus*), and one in the inferior eyelid (Figure-1). Six OSCCs affected the right eye and four the left eye. Six of 10 OSCCs were classified grossly as nodular, two were considered plaques, and two were classified as verrucous. Six animals were purebred Holstein cows, and four were Holstein crossbreeds.

**Figure-2:**
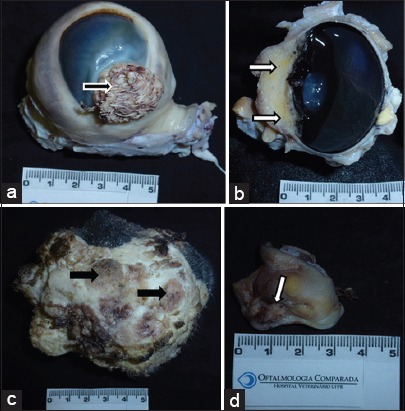
Gross pathology aspect of representative ocular squamous cell carcinoma (OSCC) cases investigated. (a) Bov17 - A conspicuously verrucous corneo conjunctival (limbal) mass of about 2.3 cm in horizontal diameter (arrow). (b) Bov15 - The tumor involved the corneal epithelium (arrows), spread to the adjacent ocular surface tissues and even invaded intraocular structures. The animal had unpigmented eyelids. (c) Bov16 - crusty and hyperemic plaque-like structures present in the inferior eyelid of the left eye (arrows). (d) Bov30- longitudinally cut the third eyelid. It is possible to see the internal cartilaginous “T” (asterisk). The arrow shows the OSCC lesion at the margin of the third eyelid.

**Figure-3:**
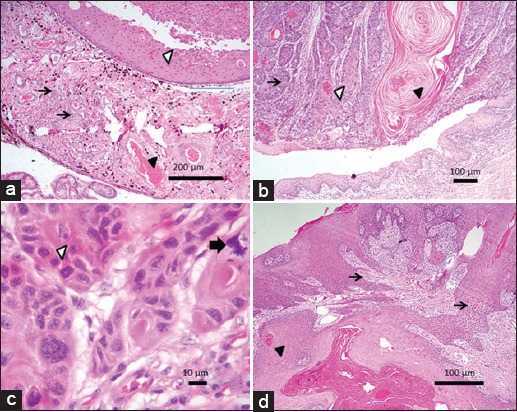
Photomicrographs of selected cases of ocular squamous cell carcinoma (OSCC) investigated. (a) Bov15 - tumor cells invaded the anterior uveal tissue forming several small islands (arrows) in the iris stroma causing severe uveitis; (b) Bov30 - third eyelid showing neoplastic cell invasion forming small islands (arrows) of keratinized cells and keratin pearls (arrowhead). Note the severe subepithelial inflammatory cell infiltrate in the adjacent conjunctiva of the third eyelid; (c) Bov16 - cellular and nuclear atypia with several mitotic figures (arrowheads). One of these mitotic figures is aberrant (arrow). Note that these cells are keratinocytes (presence of desmosomes); (d) Bov04 - inflammatory cell infiltrate with intraepithelial neoplastic corneal cell projections demonstrating dermal stroma invasion (arrows) and accentuated keratinization (keratin pearls-like structure - arrowhead).

There was a significant (p=0.044) moderate positive correlation (r=0.643) between MCF/hpf and tissue invasion. No significant, moderate, or strong correlations were found between anaplasia, invasiveness, local aggressiveness, and inflammation in the 10 OCSSs analyzed (Table-2). Only one OSCC (Bov12, Figure-1b) was highly anaplastic but had minimal invasion and low mitotic count (Table-2). Another two samples had a high mitotic count (Bov16 and 17) but low (Bov16, Figure-2c) to moderate (Bov17, Figure-2a) degrees of cellular differentiation and tissue invasion (Table-2). Similarly, the highest inflammation score observed in any sample (Bov27) did not correlate with any other high scores (Table-2). Only one OSCC had a high degree of tissue invasion (Bov15, Figures-2b and 3a), but a low degree of anaplasia.

**Figure-4:**
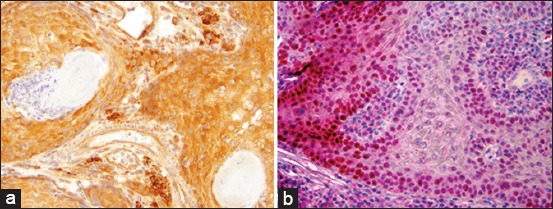
Photomicrographs of immunoexpression of p16 and p53 of ocular squamous cell carcinoma investigated. (a) Bov21 - regionally intense positive immunostaining of cytoplasmatic p16. (b) Bov17 - intense positive immunostaining of p53 with red chromogen. Note that, p53 was expressed mostly within the outer epithelial layer of the cell nests.

Three OSCCs (Bov17, Bov09, and Bov04) were positive for p53. The expression of p53 in these cases was mainly noted within the outer epithelial layer of cell nests, especially in Bov09 and Bov17 (Figure-4). The p53 LI was strong in Bov17 and focal/weak in Bov04 and Bov09. Cytoplasmic labeling for p16 was detected in three samples (Bov16, Bov21, and Bov09), while no sample had nuclear labeling (Figure-4).

PCR for generic herpesvirus and PCR or ISH for generic papillomavirus were negative for all samples.

## Discussion

The number of cases received in our study was not as high as expected based on prevalence data from other countries [1-8]. This lower number could represent a simple lack of submission of cases or truly reflect a lower prevalence. Alternatively, there are not as many OSCC cases in this Brazilian region due to a lower ultraviolet index (UVI) and other factors including genetic resistance or a lower rate of infection with oncogenic viruses.

Our results also were considerably different from one study [33] that reported 8 years as an average age of OSCC-affected cattle. In our study, 40% of our samples with the diagnosis of OSCC were from 3-year-old animals. Thus, we believe that the average age for OSCC occurrence in cattle is probably much lower than 8 years. Our results, nonetheless, corroborate another study [34], who also reported that OSCC is actually common in younger animals but rarely younger than 3 years of age.

According to our results (Table-1), the main site for OSCC was third eyelid (60%) (Figure-3b) followed by the corneo conjuntival junction (*limbus*) (20%), cornea (10%), and eyelids (10%). Our findings are similar to those by Gharagozlou *et al*. [35] that identified 70% of OSCC in the third eyelid and adjacent conjunctiva in dairy cattle. In contrast, the current results are different than Anderson *et al*. [9,10] who indicated that roughly 75% of ocular carcinomas were found on the limbus and cornea and from Pugliese *et al*. [36] who diagnosed 83.3% of the OSCC cases over the bulbar conjunctiva, cornea, and limbus. We propose that tumor location may vary according to the etiology and geographical distribution of the cattle population studied.

Our results show a tendency for high MCF/hpf to go with high tissue invasion scores (and vice versa). This was somewhat an expected result since strong positive correlations of mitotic index with other tissue invasion features such as tumor vascularization, the presence of tumor emboli in the peripheral microvessels, and compromised lymph nodes are typically observed in human head and neck SCCs [37]. No considerable correlation was found between degree anaplasia, and MCF/hpf in the OSCCs analyzed using a combination of two well-established grading systems. That is, undifferentiated OSCCs were not more invasive or possessed a higher mitotic index than well-differentiated ones. However, a high number of MCF/hpf indicated local aggressiveness. One possible explanation for these findings might be that the tumors investigated had different causes or predisposing factors, and the main histologic features conceivably vary according to the etiology. However, it is important to bear in mind that the animals were not thoroughly examined for metastasis in distant sites of the body. Thus, our results do not consider the presence of metastasis in a distant site as this was not investigated. Alternatively, the histologic grade of the SCC reflects characteristics individual neoplasm that can vary independently. Our results agree with the previous histologic investigation of OSCC [24] that could not find a clear relationship between the grade itself and cure rate, stage of disease and metastatic involvement. The presence of inflammation also varied considerably in the samples studied. One hypothesis is that inflammation was a confounding variable influenced by external factors such as conjunctivitis due to tissue exposure, presence of dirt, auto-traumatism, parasitic infestation by fly larvae (maggots) (Figure-1c), and feasibly due to the individual host response to tumor antigens [38].

Three of the investigated cases were considered positive for p16 immunoexpression. These tumors were all well differentiated with a mitotic index varying from minimal to maximal where the maximum index correlated with the strongest immunoexpression observed. However, in the majority of cases studied, p16 immunoexpression was negative. Further investigations are required to investigate p16 immunoexpression as a prognostic factor for cows with OSCC as has been previously established for humans with the same tumor in the skin [15]. We suggest evaluation of local lymph nodes may clarify these possibilities.

Immunoexpression of p53, indicating a mutation in this gene [39,40], was observed in three of 10 cases, and, considered very strong in just one case (Bov17). No clear association was found between p53 immunoexpression LI and AIM score. However, the highest AIM score was observed in a p53-positive sample concurrently with the strongest LI (Bov17). In addition, it has been shown that p53 expression in the outer epithelial layer of cell nests observed here on three cases (Bov17, Bov09, and Bov04) is correlated with invasiveness at least in oral SCCs [26,41]. It was previously demonstrated that UV radiation may initiate tumorigenesis by inducing mutations in the p53 tumor suppressor gene [42-45]. Some viral infections also can also play a role in carcinogenesis due to viral binding on p53 gene [40,43]. Even though there are Brazilian regions with much higher UVI, the animals investigated here lived in a still considerably sunny subtropical location where the UVI varies from medium (3.5 UVI) during the winter to the extreme (13.4 UVI) during the summer months [46]. Thus, it is conceivable that mutations induced by ultraviolet rays had some influence in the etiology of OSCC. The ocular location of the lesion in these three cases supports the influence of UV radiation since they were located in normally unpigmented or poorly pigmented ocular tissues (i.e., corneal, corneoconjunctival masses in two cases or affecting the third eyelid in one case). However, it was not the single most important etiological factor since the majority of the cases did not show p53 immunoexpression. Regarding pigmentation of the skin of the eyelids, two of the three positive cases had unpigmented skin, while five out of seven negative for p53 had pigmented skin. It is possible the presence of pigmented tissues provided some protection against UV damage in the population studied. Alternatively, others factors such as genetic predisposition might have played a role in the carcinogenesis. Our results differ from what was observed previous OSCC reports that demonstrated high p53 immunoexpression in a much higher percentage of cases and most animals presented skin hypopigmentation [16,46]. In our study, only one case out of 10 showed high immunoexpression of p53 (Bov17) and, in this case, the mitotic index was also high but anaplasia was mild. Thus, no association could be established between p53 immunoexpression and cancer cell differentiation which corroborates the findings from previous investigators [16].

PCR or ISH was negative for generic herpesvirus and papillomavirus in all samples. The papillomavirus was investigated because of its oncogenic potential due to its stimulus to epithelial growth, apoptosis resistance and chromosomal instability caused by viral binding and degrading of p53 protein as demonstrated in humans [47]. Additionally, detection of papillomavirus DNA was possible in several cases of SCC in cats, sheep, cattle, and horses [48-50]. In experimental studies, viral particles of bovine herpesvirus type 5 were detected in the cytoplasm of bovine OSCC after exposure to UV rays [23]. It was also discovered that p53 is an important host cell regulator for human herpesvirus replication and pathogenesis [51]. We consider the lack of herpesvirus and papillomavirus detection in the current study demonstrates that any generalized implication of these viruses playing a role in OSCC carcinogenesis continues to be problematic. In the future, with the progress of this type of investigation, different subsets of bovine OSCC will likely be identified including some with no viral association as seen with the classification of vulvar intraepithelial neoplasia in humans [52].

## Conclusion

OSCC samples were successfully characterized in this investigation. However, the etiology of remains not completely understood. Our results indicate that OSCC occurrence is most likely multifactorial in nature, with genetic, phenotypic, environmental (prolonged exposure to ultraviolet light) contributing to the pathogenesis of the disease.

It is also conceivable that the etiology may be different in the various regions of the world. It was not possible to predict local tumor behavior based on the main histological features investigated here alone. Nevertheless, we still believe that histologic classification is an important tool that needs to be studied in more detail to unveil OSCC etiology. This was a first report to produce a comprehensive histological grading scheme in a Brazilian OSCC study.

## Authors’ Contributions

GAF, FM, and DS carried out the study (diagnosis, sampling, and tissue processing). IRDBF, FM, MK, and GAF collectively planned, designed and supervised the experiment, MK and DS provided technical support and carried out the IHC, *in situ* hybridization and PCRs, FM carried out the statistical analysis. All authors read and approved the final manuscript.
